# Hepcidin in hospitalized older medical patients: associations with inflammation, anemia, frailty, comorbidity, and renal function

**DOI:** 10.1186/s12877-026-07638-w

**Published:** 2026-05-19

**Authors:** Louis Praeger-Jahnsen, Martin Aasbrenn, Rikke S. Kamper, Martin Schultz, Pernille Hansen, Sofie K. Hansen, Eckart Pressel, Jens Rasmussen, Mikkel Bring Christensen, Sisse B. Ditlev, Charlotte Suetta, Hanne Nygaard

**Affiliations:** 1 Copenhagen Center for Translational Research, Copenhagen University Hospital - Bispebjerg and Frederiksberg, Copenhagen, Denmark; 2 Geriatric Research Unit, Department of Geriatric and Palliative Medicine, Copenhagen University Hospital - Bispebjerg and Frederiksberg, Copenhagen, Denmark; 3 Department of Clinical Medicine, Faculty of Health and Medical Sciences, University of Copenhagen, Copenhagen, Denmark; 4 Department of Geriatric and Palliative Medicine, Copenhagen University Hospital - Amager and Hvidovre, Copenhagen, Denmark; 5 Department of Emergency Medicine, Copenhagen University Hospital - Bispebjerg and Frederiksberg Hospital, Copenhagen, Denmark; 6 Department of Clinical Pharmacology, Copenhagen University Hospital - Bispebjerg and Frederiksberg, Copenhagen, Denmark

**Keywords:** Geriatrics, Frailty, Chronic disease, Kidney function, Iron metabolism, Acute hospitalization

## Abstract

**Background:**

Anemia is highly prevalent among hospitalized older adults and is associated with increased morbidity and mortality. Hepcidin is a liver-derived hormone that reduces iron availability and contributes to anemia. As the key regulator of iron metabolism, hepcidin is hypothesized to promote anemia development through inflammation-mediated pathways. However, the role of hepcidin in different types of anemia among frail, acutely hospitalized older patients remains poorly understood. The present study aimed to investigate the relationship between circulating hepcidin levels and markers of inflammation, anemia, frailty, comorbidity, and kidney function in a cohort of acutely hospitalized older patients.

**Methods:**

Data from 1,030 patients aged ≥ 65 years from the Copenhagen PROTECT study were analyzed. Plasma hepcidin and interleukin-6 (IL-6) were measured using immunoassays, while standard biochemical markers, including hemoglobin and C-reactive protein (CRP), were assessed by routine clinical platforms. Anemia was defined according to WHO criteria. Frailty and comorbidity were evaluated using the Clinical Frailty Scale and the age-adjusted Charlson Comorbidity Index (CCI), respectively. Kidney function was determined by estimated glomerular filtration rate (eGFR). Correlations were examined using Pearson or Spearman methods depending on distribution and post hoc analyses included subgroup comparisons stratified by frailty and CCI strata. In exploratory multivariable analyses, hemoglobin was associated with hepcidin, inflammation, renal function, frailty, and comorbidity.

**Results:**

Anemia was present in 48% of patients, with 10% of these displaying moderate-to-severe anemia. Hepcidin levels were significantly lower in patients with severe anemia compared to non-anemic patients. Notably, no consistent correlation was found between hepcidin and hemoglobin overall. Median hepcidin was 60.1 ng/mL [IQR 21.8–126.2] and showed moderate positive correlations with CRP (*r* = 0.48) and IL-6 (*r* = 0.47) (both *p* < 0.001). Hepcidin levels were lower in frail patients and varied modestly across comorbidity strata.

**Conclusions:**

In this cohort of acutely admitted older medical patients, hepcidin was moderately associated with inflammation and showed modest associations with frailty, comorbidity, and anemia severity. Although no overall correlation was found between hepcidin and hemoglobin, patients with more severe anemia showed lower hepcidin levels. These findings suggest that inflammation is the primary driver of hepcidin regulation in this population.

**Trial registration:**

The study was approved by the Regional Ethics Committee of Copenhagen and Frederiksberg (H19039214) and the Danish Data Protection Agency (P2019239).

## Introduction

Iron levels in the human body are tightly regulated, with the peptide hormone hepcidin serving as the primary controller of iron transport [1]. Hepcidin is synthesized in the liver and works by binding to the iron export protein ferroportin [2]. Hepcidin leads to the degradation of ferroportin, and through this mechanism, it decreases the circulating iron concentration, reduces dietary absorption of iron, and inhibits the release of iron from cellular storage [3, 4]. Hepcidin reduces the amount of iron available in the circulation, which subsequently can lead to a reduction in erythropoiesis and thus anemia [5].

Multiple factors can lead to the release of hepcidin. Iron intake induces hepcidin expression, thus reducing dietary absorption of iron [6]. In contrast, conditions such as hypoxia and anemia downregulate hepcidin, thereby enhancing iron uptake for erythropoiesis [7]. Furthermore, inflammatory stimuli such as Interleukin-6 (IL-6) can induce the expression of hepcidin [4, 8, 9]. Impaired kidney function also increases the production of hepcidin and thus may contribute to the development of anemia in older patients [10].

Anemia is highly prevalent among hospitalized older patients, particularly those with frailty and chronic illnesses [11, 12], and iron deficiency anemia, followed by anemia of inflammation represents the most common subtypes observed in this patient population [13]. It has been hypothesized that activation of the hepcidin-axis may play a crucial role in anemia of inflammation [9]. Prior studies state that chronic inflammation leads to increased hepcidin synthesis, contributing to anemia from inflammation [5, 14–17]​. However, little is known about hepcidin regulation and its impact during acute inflammation and disease, particularly in frail, older patients.

Among the older patients, the risk of readmission and death following hospitalization is increased [18, 19]. Additionally, they have a high risk of losing mobility and becoming dependent on others for daily activities, which leads to higher healthcare costs [20]. Anemia may be implicated in the occurrence of these serious outcomes after admission, as reduced oxygen delivery and increased cardiac strain can compromise recovery, and underlying comorbidities may further worsen prognosis [21]. Thus, a better understanding of the underlying causes of anemia in this expanding patient population is needed.

Hepcidin has considerable clinical interest in this context. If hepcidin activation contributes to some types of anemia in hospitalized patients, therapeutic interventions targeting the hepcidin axis may warrant investigation in relevant subgroups. Because conventional iron markers, especially ferritin, are strongly influenced by acute-phase reactions, hepcidin may help to more accurately distinguish anemia subtypes in acutely ill older patients [22]. This study aims to investigate the relationships between hepcidin and frailty, inflammation, anemia, and renal function in a cohort of acutely admitted older medical patients.

## Methods

The study is reported in accordance with the Strobe Guidelines [23].

### Aim

The present study aimed to investigate the relationship between circulating hepcidin levels and markers of inflammation, anemia, frailty, comorbidity, and kidney function in a cohort of acutely hospitalized older medical patients.

### Study design

The present study was designed as a secondary analysis of cross-sectional data.

### Setting

All included patients were admitted to the acute medical ward at Copenhagen University Hospital, Bispebjerg and Frederiksberg, Copenhagen, Denmark. The patients were enrolled within the first 24 h following their admission. Patients were recruited from November 2019 to November 2021 with minor pauses during the COVID-19 pandemic [24].

### Participants

Eligible patients were acutely admitted patients aged ≥65years. Exclusion criteria were admission duration >24 h at the time of baseline assessment, terminal illness, temporary social security number (foreign citizens), infections requiring droplet or airborne isolation, being medically unsuitable for inclusion, inability to speak or read Danish, or otherwise not able to provide informed consent. Additional information can be found in the study protocol [24]. Their initial admission was recorded as the index admission. The trial was conducted in accordance with the Declaration of Helsinki, and all patients provided informed consent.

### Study size

The study sample size was based on the Copenhagen PROTECT cohort, in which 1,071 patients were originally enrolled. Of these, 1,030 had complete data available for hepcidin analysis and were included in the present study. These patients were categorized based on hemoglobin levels (anemia), eGFR (Kidney function), CFS (frailty), CCI (comorbidity score), age, and sex. The cohort has previously been characterized in relation to frailty and clinical outcomes [25].

### Variables and data source

A broad set of demographic and clinical variables were collected according to the Copenhagen PROTECT study protocol [24]. Age, sex, and medical diagnoses were obtained from the electronic medical record system (Sundhedsplatformen, Epic^®^). Admission diagnoses were grouped into predefined diagnostic categories and used to describe the clinical context of the index admission; the distribution of these diagnostic categories is presented in Table 1. Each diagnostic category comprises several specific diagnosis groups; a detailed overview of the categories and their constituent diagnosis groups is provided in Appendix 1. Comorbidity was quantified using the age-adjusted Charlson Comorbidity Index (CCI), calculated based on ICD-10 diagnosis codes available at baseline admission [24, 26].

Frailty was assessed as part of the clinical baseline assessment within 24 h of admission, using the Clinical Frailty Scale (CFS) [27] as described in the PROTECT study protocol. All frailty assessments were performed by trained personnel in accordance with standardized procedures.

Routine biochemical data included hemoglobin, albumin, creatinine, and CRP were analyzed at the Department of Clinical Biochemistry, Bispebjerg and Frederiksberg Hospital. These were part of the standard admission panel and reflect real-time clinical values.

Anemia was defined according to WHO criteria: hemoglobin < 120 g/L (< 7.45 mmol/L) for non-pregnant females and < 130 g/L (< 8.07 mmol/L) for males. Anemia severity was categorized as mild (Hb 110–119 g/L for female / 110–129 g/L for male), moderate (Hb 80–109 g/L), or severe (< 80 g/L) for both sexes [28]. Frailty status was dichotomized as “non-frail” (CFS < 5) and “frail” (CFS ≥ 5) [29]. Comorbidity was categorized based on CCI into low (1–4), moderate (5–8), and high (9–14) [30]. Kidney function was estimated by eGFR (CKD-EPI) and grouped into normal (≥ 90), mildly decreased (60–89), moderately decreased (30–59), and severely decreased (< 30 mL/min/1.73 m²) [4, 31]. CRP > 10 mg/L and IL-6 > 6 pg/mL were defined as elevated [32–34].

### Project specific biomarkers

Blood samples were drawn from the antecubital vein after an overnight fast and collected in serum and EDTA tubes, respectively. Samples were kept on ice and centrifuged within 1 h at 2500 g for 10 min at 4 °C. Plasma and serum aliquots were subsequently stored at − 80 °C until analysis. Hepcidin and IL-6 were analyzed using the stored biobank samples. IL-6 was measured using a V-PLEX multiplex assay (Mesoscale Discovery) on plasma samples with a 2-fold dilution. Hepcidin was measured on serum samples using a U-PLEX assay (Mesoscale Discovery) after pre-testing to determine the optimal 20-fold dilution, in accordance with the protocol. All measurements were performed on a MESO QuickPlex SQ 120 instrument (Meso Scale Discovery).

### Statistical analysis

Data are presented as mean (standard deviation (SD)), median (interquartile range (IQR)), or proportion depending on the distribution of the data. Correlations were visualized in plots and assessed with the Pearson’s or Spearman’s correlation coefficients according to the type and distribution of data. In post hoc analyses, a multiple linear regression analysis with hemoglobin (mmol/L) as dependent variable was performed to clarify relative strength between independent variables. Model testing was initiated and log transformation applied where relevant to address skewness and deviations from normality assumptions. Variables reflecting inflammation (CRP, IL-6), renal function (eGFR), frailty (Clinical Frailty Scale), comorbidity burden (Charlson Comorbidity Index), and hepcidin were included based on clinical relevance. Backward elimination was used, eliminating sex and age from the final model. The overall model F-test and adjusted R-Square, in addition to parameter and standardized estimate for each independent variable was calculated. In addition, the association between hepcidin and anemia was explored across levels of frailty and comorbidity. Non-parametric group comparisons were performed using Wilcoxon rank-sum tests for pairwise comparisons, and a Kruskal–Wallis test was applied to assess differences in hepcidin levels across the three comorbidity groups. For descriptive purposes, age was stratified into 5-year intervals but treated as a continuous variable in all primary analyses [26]. All statistical analyses were performed using R (version 4.4.2) within RStudio (version 2024.04.2; Posit Software, PBC). A two-tailed significance level of *p* < 0.05 was considered statistically significant for all tests.

## Results

The mean age of the included patients was 78 ± 7.8 years, and 52.8% (*n* = 544) were females. Totally, 48.4% (*n* = 499) were classified as frail (CFS ≥ 5), and 57.9% (*n* = 597) had moderate to high comorbidity (CCI ≥ 5).

Among the included patients, 61% (*n* = 629) had elevated CRP levels, and 52% (*n* = 534) exhibited elevated IL-6 levels.

More than half of the cohort had some degree of anemia: 41.6% (*n* = 428) had mild anemia and 10.0% (*n* = 103) had moderate-to-severe anemia (Table 1). A substantial proportion of the patients were admitted with diagnoses classified as infectious diseases, accounting for approximately 38–41% across anemia severity groups (Table 1). As detailed in Appendix 1, this category primarily comprised acute infections such as pneumonia, sepsis, urinary tract infection, and infections of unknown focus, reflecting that the patients were recruited at an acute medical ward.

**Table 1:** Population characteristics and summary of variables across anemia status groups

Variable	Total	No Anemia	Mild Anemia	Moderate to Severe Anemia
Total n (%)	1030	499 (48.4%)	428 (41.6%)	103 (10.0%)
Female n (%)	544	216 (39.7%)	277 (50.9%)	51 (9.4%)
Male n (%)	486	283 (58.2%)	151 (31.1%)	52 (10.7%)
Age (years)	78 [73–85]	77 [72–84]	80 [74–86]	79 [72–85]
eGFR,^d^ (mL/min/1.73 m²)	66 [46–83]	72 [52–85]	61 [41–79]	57 [34–80]
CFS^b^	4.0 [3.0–5.0]	4.0 [3.0–5.0]	5.0 [4.0–6.0]	5.0 [4.0–6.0]
CCI^a^	5.0 [4.0–6.0]	5.0 [4.0–6.0]	5.0 [4.0–6.0]	5.0 [5.0–7.5]
Albumin (g/L)	32 [28–35]	33 [30–36]	31 [27–34]	26 [22–31]
CRP^c^ (mg/L)	27 [5–98]	23 [4–86]	31 [5–107]	49 [8–108]
Hemoglobin (g/L)	126 [111–139]	137 [130–147]	118 [114–122]	98 [89–105]
Hepcidin (ng/mL)	60.1 [21.8–126.2]	61.7 [28.1–122.8]	63.2 [20.4–129.3]	37.9 [2.0–94.6]
IL-6^e^ (pg/mL)	6.9 [3.0–16.8]	6.3 [2.7–14.6]	6.9 [3.4–18.2]	9.7 [3.9–20.0]
Creatinine (µmol/L)	83 [66–114]	81 [65–105]	85 [67–122]	93 [70–138]
LDH^f^ (U/L)	202 [173–243]	203 [174–250]	203 [172–241]	198 [169–238]
Infectious diseases	401 (38.9%)	204 (38.1%)	92 (38.2%)	105 (41.5%)
R diagnoses (symptoms & abnormal findings)	163 (15.8%)	87 (16.2%)	52 (21.6%)	24 (9.5%)
Respiratory diseases	95 (9.2%)	75 (14.0%)	14 (5.8%)	6 (2.4%)
Endocrine and nutritional diseases	92 (8.9%)	45 (8.4%)	24 (10.0%)	23 (9.1%)
Digestive diseases	61 (5.9%)	20 (3.7%)	17 (7.1%)	24 (9.5%)
Musculoskeletal diseases	58 (5.6%)	32 (6.0%)	15 (6.2%)	11 (4.3%)
Other	59 (5.7%)	25 (4.7%)	6 (2.5%)	28 (11.1%)
Mental and behavioral disorders	38 (3.7%)	25 (4.7%)	10 (4.1%)	3 (1.2%)
Cardiovascular diseases	29 (2.8%)	14 (2.6%)	5 (2.1%)	10 (4.0%)
Hematological diseases	16 (1.6%)	0	0	16 (6.3%)
Neurological diseases	10 (1.0%)	6 (1.1%)	3 (1.2%)	1 (0.4%)
Z diagnoses (factors influencing health status)	8 (0.8%)	3 (0.6%)	3 (1.2%)	2 (0.8%)

Data are given as median [interquartile range]. Sex and diagnosis categories are presented as n (%). Sex-specific percentages are calculated across anaemia categories, whereas diagnosis-specific percentages are calculated within each anaemia category. The number of available observations varied across laboratory variables: Albumin (*n* = 1012), CRP (*n* = 1021), IL-6 (*n* = 1027), LDH (*n* = 755), Creatinine (*n* = 1026), eGFR (*n* = 1027), and Hepcidin (*n* = 1032). For definitions of the diagnosis categories, see Appendix 1

^a^CCI – Charlson Comorbidity Index; ^b^CFS – Clinical Frailty Score; ^c^CRP – C-reactive protein; ^d^eGFR – estimated glomerular filtration rate; ^e^IL-6 – interleukin-6; ^f^LDH – lactate dehydrogenase

With the increasing severity of the anemia, several biomarkers show variation. Markers of inflammation as CRP and IL-6, increase with anemia severity (CRP: 23 mg/L [4–86] in no anemia vs. 49 mg/L [8–108] in moderate-to-severe anemia; IL-6: 6.3 pg/mL [2.7–14.6] vs. 9.7 pg/mL [3.9–20.0]). Hepcidin concentrations were lowest in the most severe anemia group (37.9 ng/mL [2.0–94.6]).

CFS increased with anemia severity, from a median of 4 [IQR: 3–5] in patients without anemia to 5 [IQR: 4–6] in those with mild to moderate-to-severe anemia. Similarly, the CCI median remained at 5 across all anemia groups, with an interquartile range of 4–6 in patients with no or mild anemia and 5–7.5 in those with moderate-to-severe anemia.

**Fig. 1:**
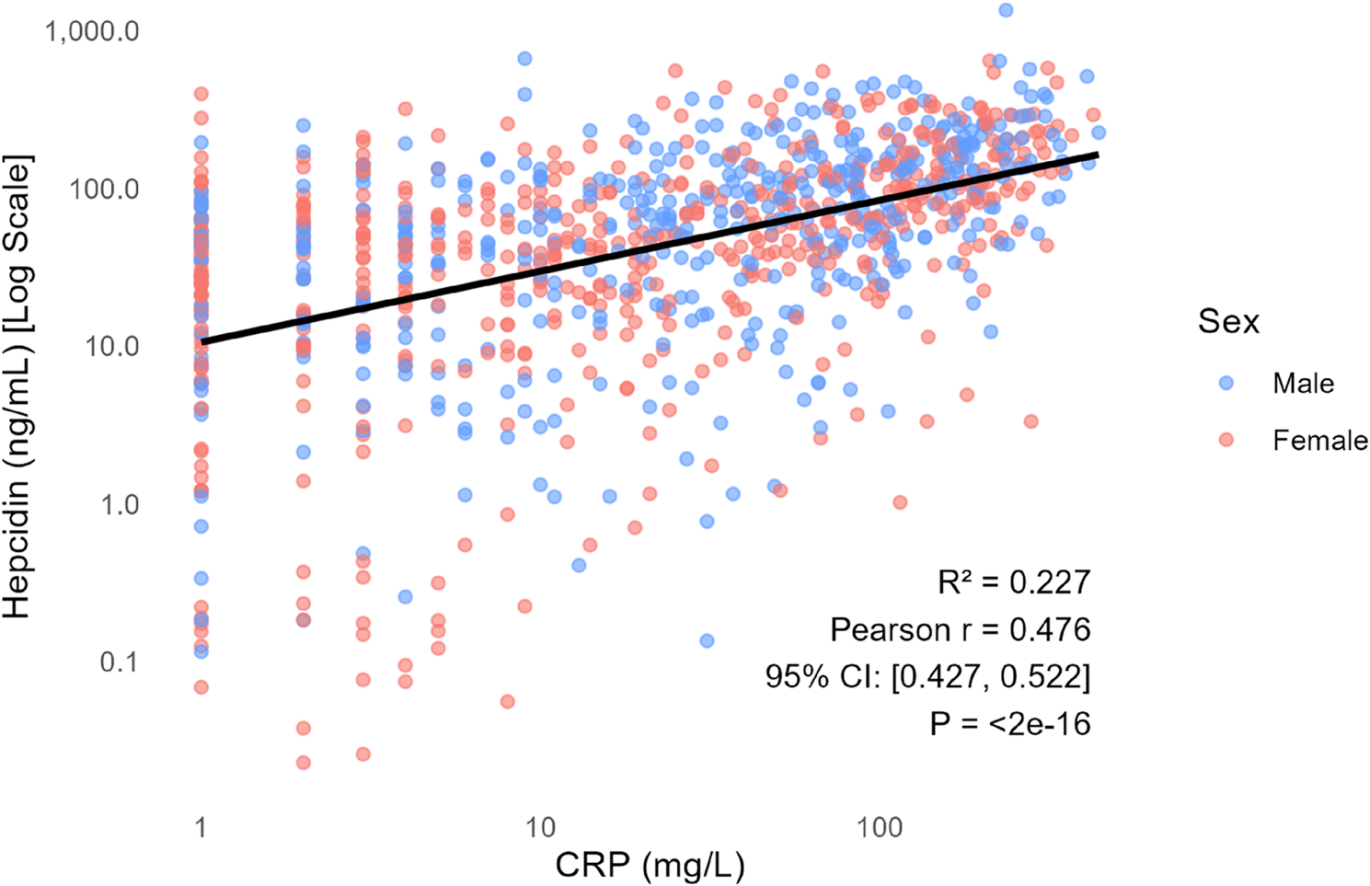
The association between hepcidin concentrations (ng/mL) and CRP (mg/L) concentrations. The association between hepcidin concentrations (ng/mL) and CRP (C-reactive protein) concentration (mg/L). The x-axis shows a logarithmic CRP level, and the y-axis shows a logarithmic hepcidin level. The black linear regression trend line (R² = 0.227) depicts the relation between the two variables

The scatterplot in Fig. 1 illustrates the relationship between hepcidin and CRP concentrations. The black regression line indicates a positive correlation between CRP and hepcidin concentrations, with Pearson’s correlation yielding a coefficient of 0.476 (95% CI 0.427–0.522, *p* < 0.0001). The model explained 22.7% of the variance (R²=0.227), indicating a moderately strong correlation.

**Fig. 2:**
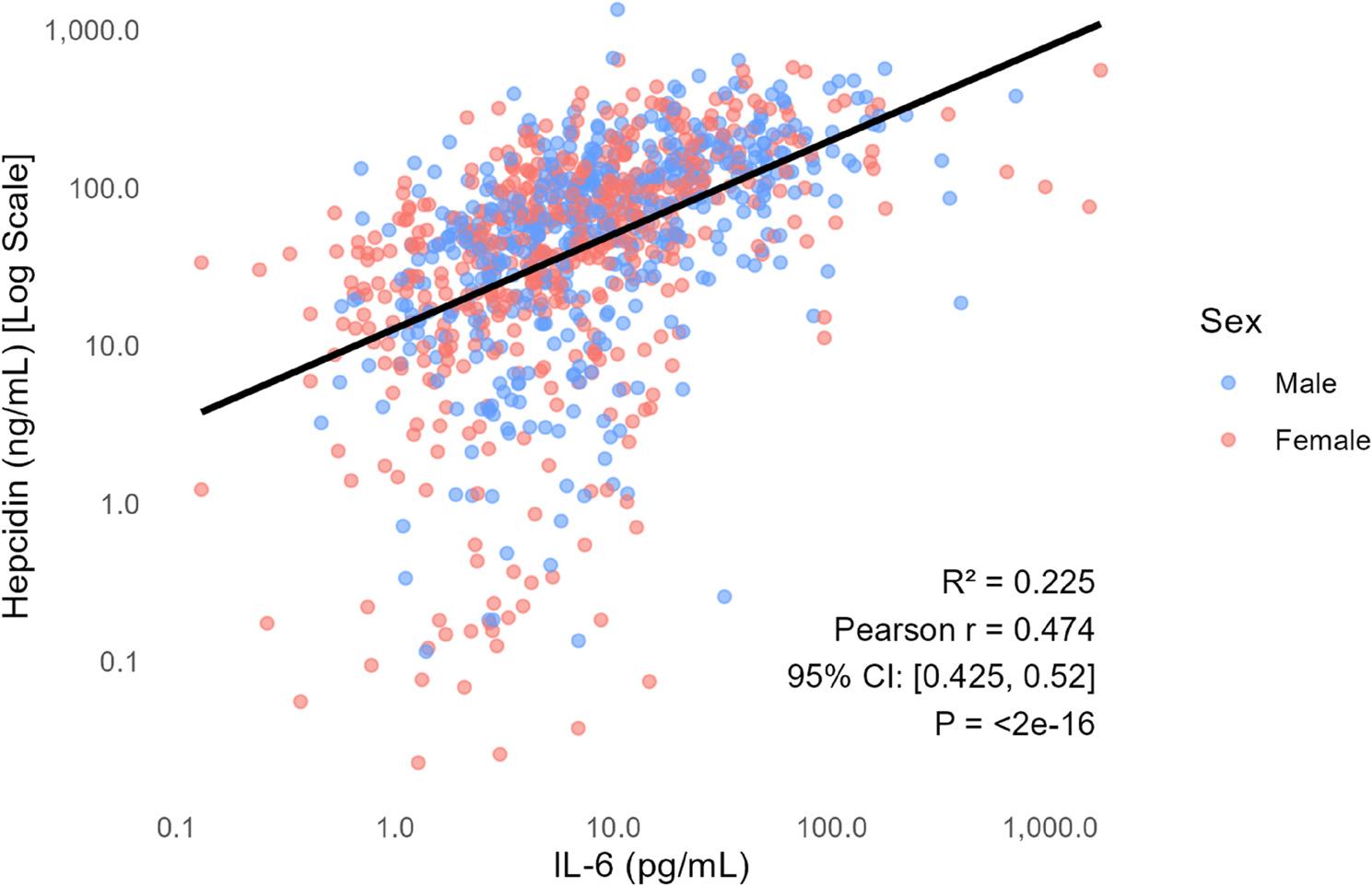
The association between hepcidin concentrations (ng/mL) and IL-6 (pg/mL) concentrations. The association between hepcidin concentrations (ng/mL) and IL-6 (pg/L) concentrations. The x-axis shows a logarithmic IL-6 concentration, and the y-axis shows a logarithmic hepcidin concentration. The black linear regression trend line (R² = 0.225) depicts the relation between the two variables

Figure 2 displays a scatterplot illustrating the relationship between hepcidin and IL-6 concentrations. The black regression line indicates a positive correlation between IL-6 and hepcidin concentrations, with Pearson’s analysis yielding a coefficient of 0.474 (95% CI 0.425–0.520, *p* < 0.001). The model explained 22.5% of the variance (R²=0.225), indicating a moderately strong correlation.

**Fig. 3:**
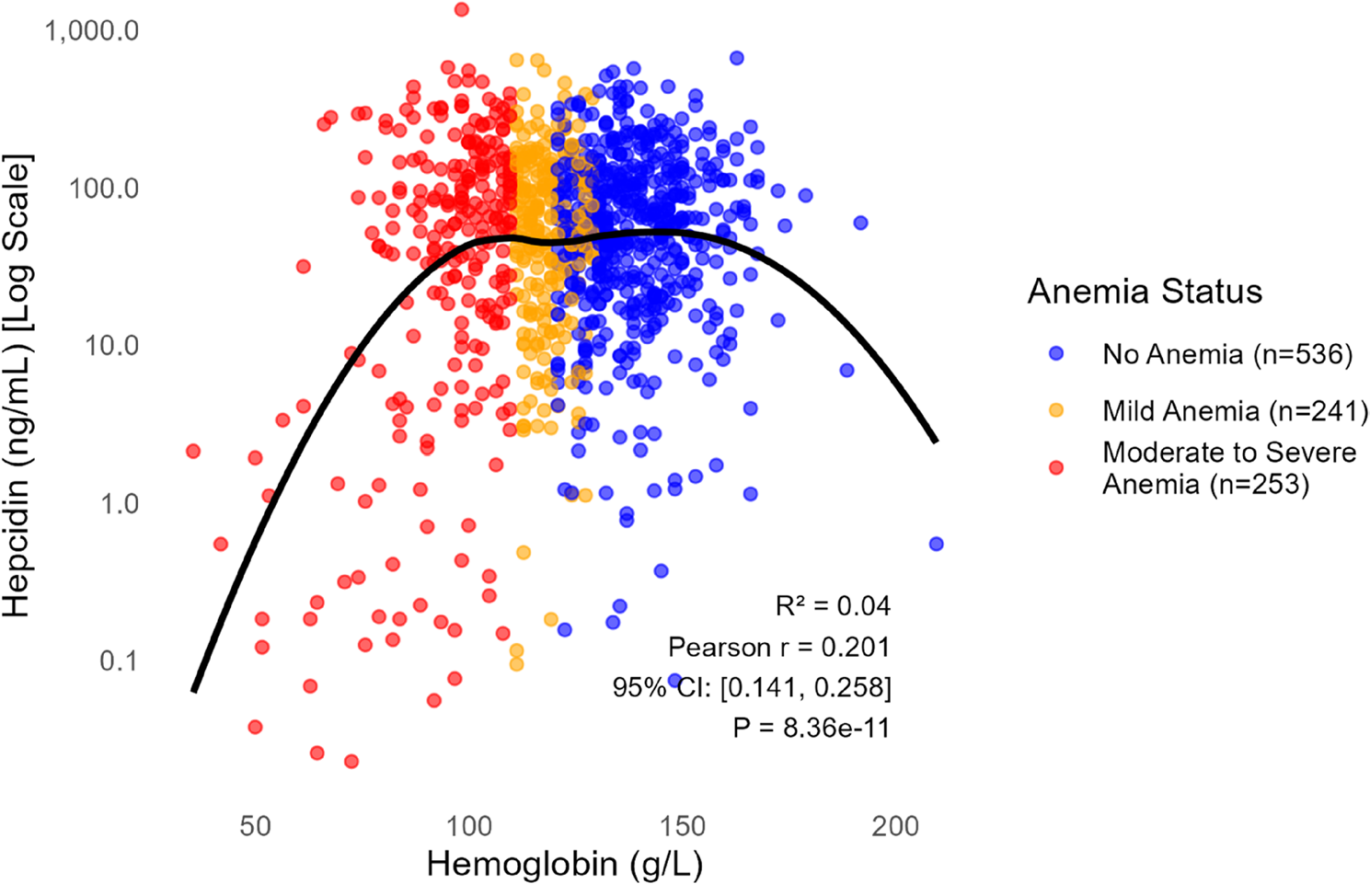
The association between hepcidin concentration (ng/mL) and hemoglobin concentrations (g/L). The association between hepcidin concentration (ng/mL) and hemoglobin (mmol/L) concentrations. The x-axis shows linear hemoglobin concentration, and the y-axis shows a logarithmic hepcidin concentration. The read linear regression trend line (R² = 0.04) depicts the relation between the two variables

Figure 3 shows a scatterplot with hepcidin and hemoglobin. The R² values were calculated separately for each anemia group. No anemia had an R² of 0.0050, mild anemia was found to have an R² of 0.0025, and moderate-to-severe anemia had an R² of 0.2199. The overall R² of hepcidin vs. hemoglobin was found to be 0.0402 with a Pearson *r* = 0.201 (95% CI 0.141–0.258, *p* < 0.0001). The slight overlap between mild and no anaemia reflects the different haemoglobin cut-off values for females (< 120 g/L) and males (< 130 g/L).

**Fig. 4:**
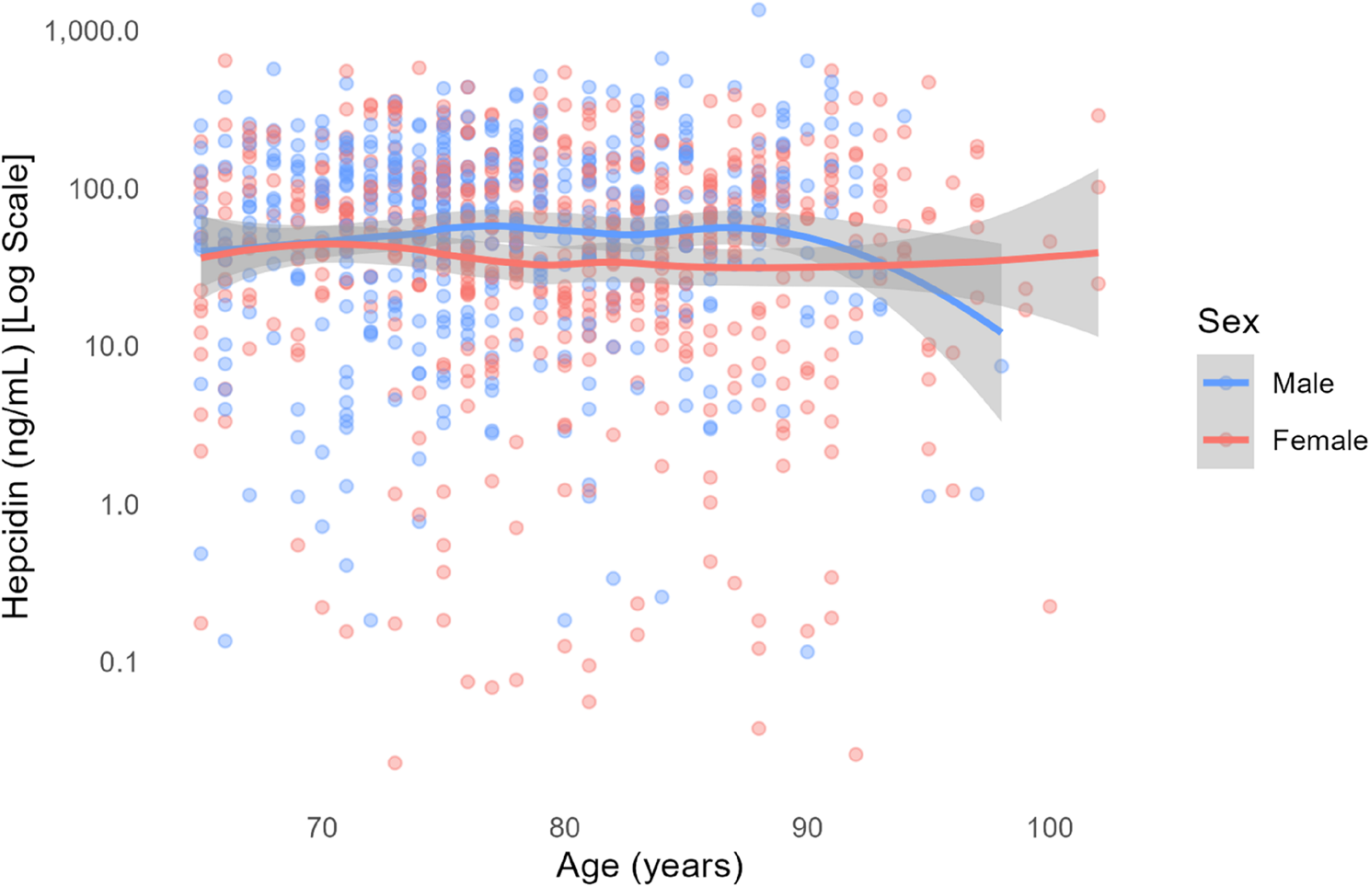
The association between hepcidin concentration (ng/mL), sex, and age. Illustrates the hepcidin distribution across age and sex groups (female = red, male = blue). Age groups are divided into 5-year increments

Figure 4 illustrates individual hepcidin values for each sex across age groups, with trend lines indicating age-related changes. Females had a mean hepcidin level of 84.6 ng/mL in the 65–70 age group (*n* = 56), 105.0 ng/mL in the 70–75 group (*n* = 88), 81.4 ng/mL in the 75–80 group (*n* = 126), 76.3 ng/mL in the 80–85 group (*n* = 110), 73.8 ng/mL in the 85–90 group (*n* = 94), 98.7 ng/mL in the 90–95 group (*n* = 48), and 71.7 ng/mL in the 95–100 group (*n* = 20). Males had a mean hepcidin level of 93.9 ng/mL in the 65–70 age group (*n* = 68), 91.7 ng/mL in the 70–75 group (*n* = 108), 108.7 ng/mL in the 75–80 group (*n* = 124), 99.7 ng/mL in the 80–85 group (*n* = 85), 114.6 ng/mL in the 85–90 group (*n* = 73), 136.5 ng/mL in the 90–95 group (*n* = 26), and 3.2 ng/mL in the 95–100 group (*n* = 3). Mean hepcidin levels were higher in male than in female patients across all age groups, except in the 70–75-year and > 90-year groups (see Fig. 4).

### Post-Hoc analyses

**Fig. 5:**
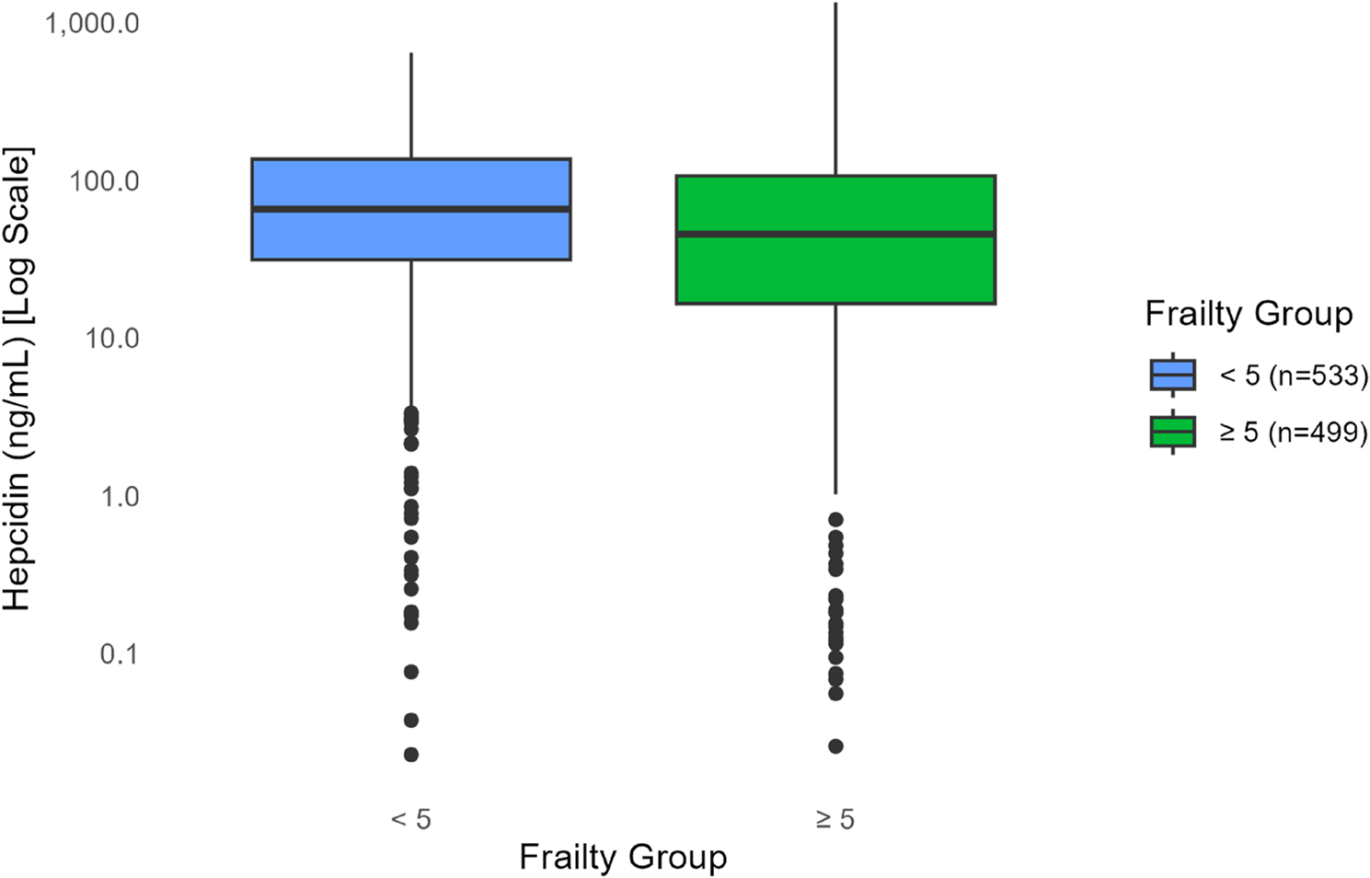
Boxplot displaying hepcidin concentration (ng/mL) in non-frail group vs. frail group (CFS). The boxplots display hepcidin concentrations variation in the non-frail vs. the frail group. Non-frail was defined as a CFS <5 (*n*=533) and frail as CSF ≥5 (*n*=499)

Figure 5 shows a modest but statistically significant difference in hepcidin concentrations between frail and non-frail participants, with higher median values observed in the non-frail group (65.96 ng/mL [31.44–136.99]) compared with the frail group (45.71 ng/mL [16.57–107.24]) (Wilcoxon rank-sum test, *p* < 0.0001).

**Fig. 6:**
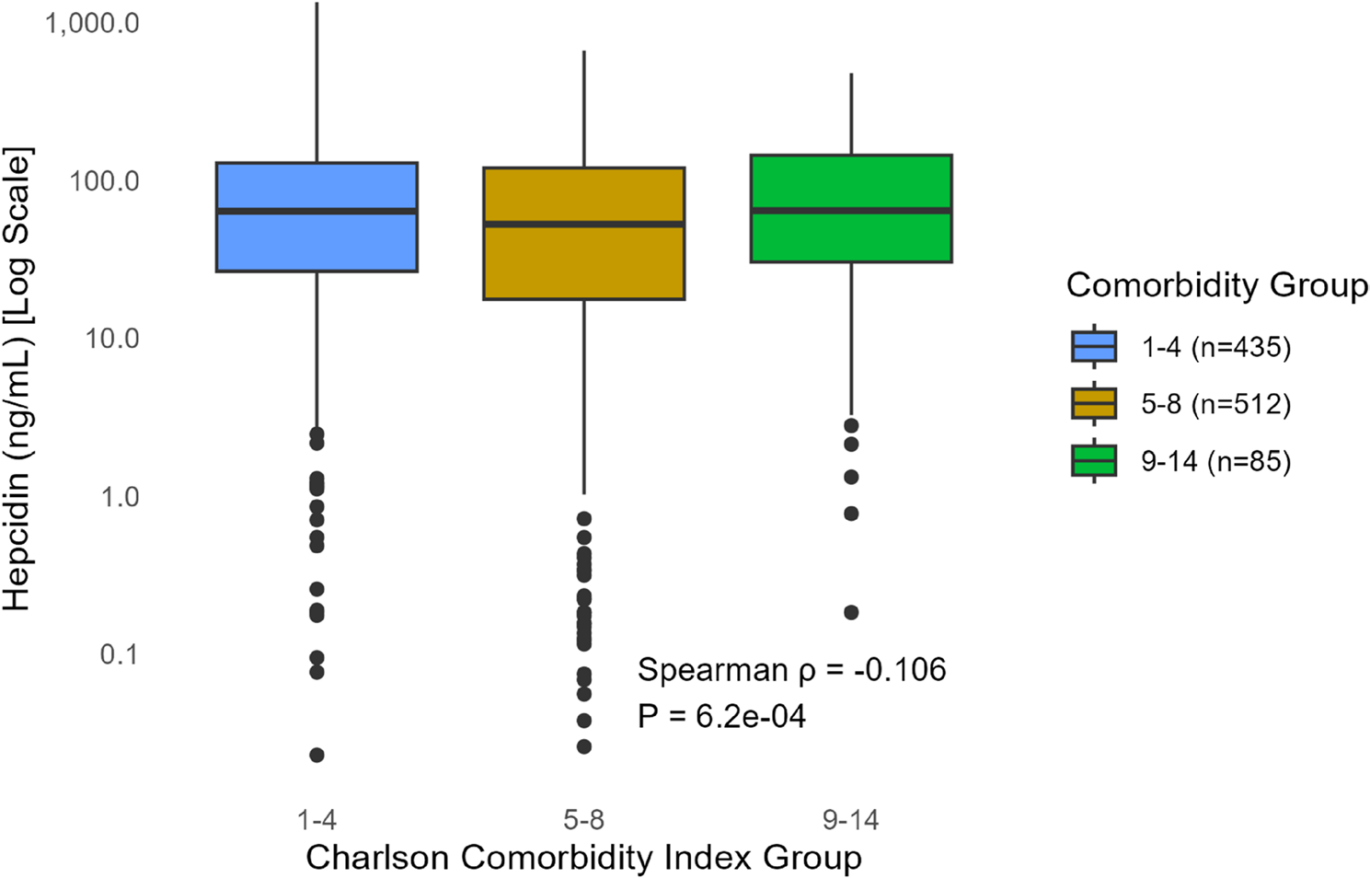
Boxplot displaying hepcidin concentration (ng/mL) across CCI groups (Charlson Comorbidity Index). The boxplots show hepcidin concentrations across Charlson Comorbidity Index (CCI) groups

Figure 6 shows no clear trend in hepcidin levels across the three CCI groups. Median hepcidin concentrations were similar in the 1–4 group (64.1 ng/mL [26.6–129.3]) and the 9–14 group (64.5 ng/mL [30.4–144.6]), while the 5–8 group had a slightly lower median of 52.9 ng/mL [17.7–120.2]. An overall comparison using the Kruskal–Wallis test indicated a statistically significant difference in hepcidin levels between the three comorbidity groups (χ² = 7.43, df = 2, *p* = 0.024). Pairwise post hoc Wilcoxon tests showed a significant difference in hepcidin levels between patients with moderate comorbidity (CCI 5–8) and those with low comorbidity (CCI 1–4; *p* = 0.008), whereas no significant differences were found between the high comorbidity group (CCI 9–14) and either the low (*p* = 0.82) or moderate (*p* = 0.22) groups.

**Fig. 7:**
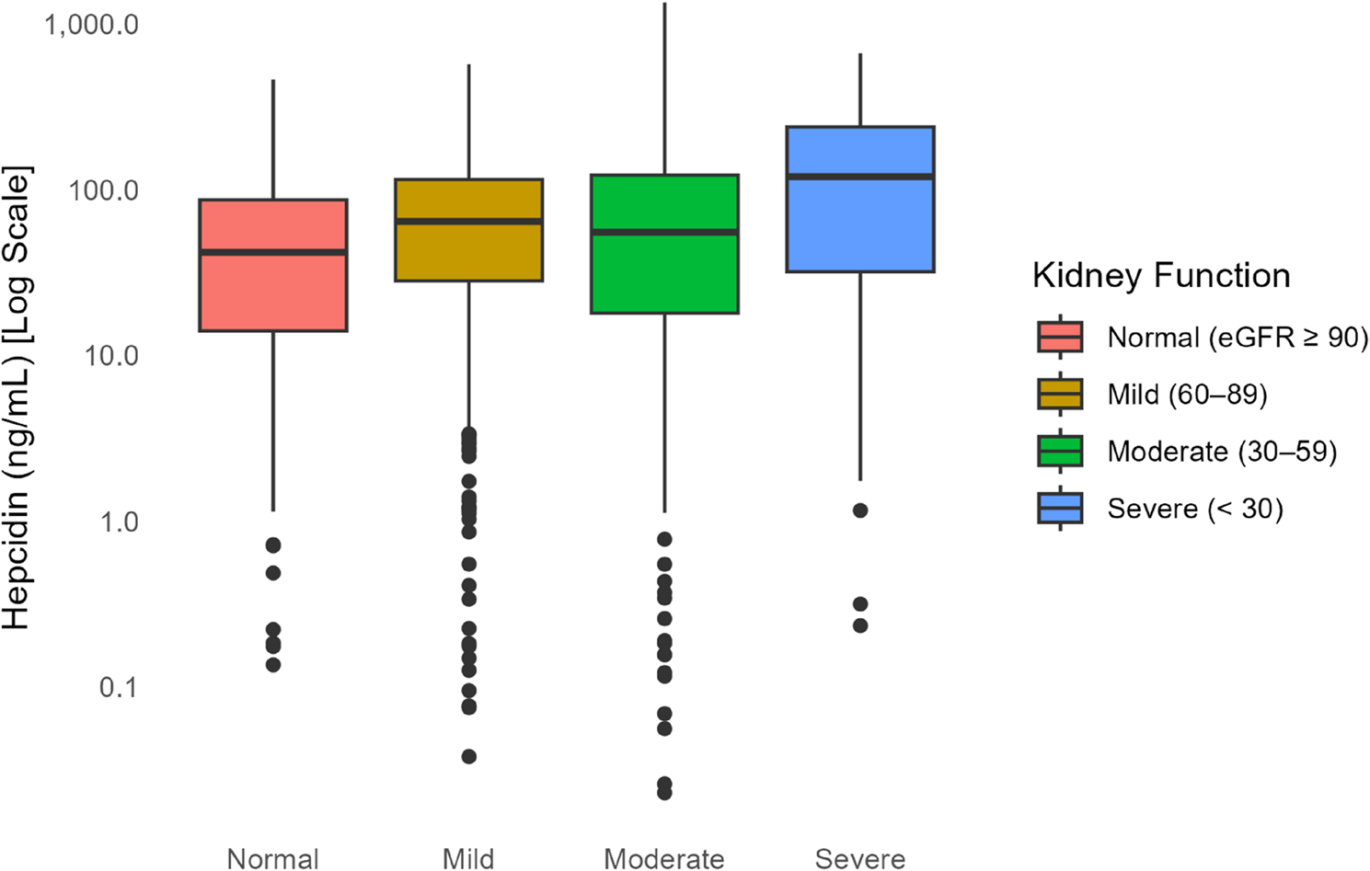
Boxplot displaying hepcidin concentration (ng/mL) across kidney function groups. Kidney function was evaluated based on eGFR and categorized into four groups: Normal (eGFR ≥ 90, *n* = 143), Mildly Decreased (eGFR 60–89, *n* = 465), Moderately Decreased (eGFR 30–59, *n* = 313), and Severely Decreased/Kidney Failure (eGFR < 30, *n* = 106)

Hepcidin concentrations were 41.8 ng/mL [14.0–86.7] in patients with normal kidney function, 64.3 ng/mL [28.1–115.0] with mildly decreased kidney function, 55.2 ng/mL [17.9–122.6] with moderately decreased function, and 120.0 ng/mL [31.9–239.1] in those with severely decreased or kidney failure (Fig. 7).

The Spearman correlation analyses across six groups stratified by comorbidity and frailty revealed no statistically significant associations between hepcidin and hemoglobin (*p* > 0.05). The highest absolute correlation was observed in the high comorbidity, non-frailty group (ρ=-0.264, *p* = 0.104). There were no significant associations between hepcidin and hemoglobin across the remaining groups, all *p* > 0.05 (ρ = − 0.18 to 0.08). Patient distribution across comorbidity and frailty strata is presented in Appendix Table 1, illustrating the relatively small sample size in the high comorbidity, low frailty group, which may have limited statistical power.

In a multivariable linear regression analysis including markers of inflammation (CRP, IL-6), renal function (eGFR), frailty (CFS), comorbidity burden (CCI), and hepcidin, the overall model was statistically significant (*p* < 0.0001) and explained approximately 19% of the variance in hemoglobin levels (adjusted R²=0.19). Higher hepcidin levels were independently associated with higher hemoglobin, whereas higher CRP and IL-6 levels were associated with lower hemoglobin. eGFR was positively associated with hemoglobin, consistent with better renal function being linked to higher hemoglobin levels. Greater CFS and CCI were negatively associated (Appendix 3). The magnitude of associations, assessed by standardized coefficients, was greatest for hepcidin and IL-6. Stratified analyses by infection status showed similar overall patterns, although effect sizes appeared attenuated among patients with infection.

## Discussion

In the present study of acutely hospitalized older medical patients, a moderate association was observed between hepcidin and inflammatory markers. Severe anemia appeared to be associated with lower circulating hepcidin, while no significant association with age or sex were identified. However, hepcidin levels were significantly lower among frail patients and varied across comorbidity levels, showing a weak negative correlation.

In acutely hospitalized older patients IL-6 is a well-established inducer of hepatic hepcidin expression. The observed association between IL-6 and hepcidin in the present study is consistent with this regulatory pathway [35, 36]. This inflammatory activation is consistent with mechanisms known to be involved in hypoferremia and anemia of inflammation [36]. In contrast, population-based data from the InCHIANTI study showed that in community-dwelling older adults with only mild, chronic inflammation, urinary hepcidin was not elevated and showed no correlation with IL-6 or CRP, despite their association with lower serum iron [15].

These findings indicate that the association between inflammation and hepcidin may differ according to clinical context, such as acute versus chronic inflammatory states.

A considerable proportion of patients were admitted with acute infectious diagnoses, as reflected by the high prevalence of infectious disease categories in Table 1 and detailed in Appendix 1. Acute infection may induce a rapid inflammatory response with hepcidin upregulation before a measurable decline in hemoglobin occurs, which could partly explain the modest and inconsistent association between hepcidin and hemoglobin observed in the overall cohort. However, the cross-sectional design and lack of temporal information on symptom onset preclude differentiation between acute, subacute, and chronic inflammatory states at admission, and bleeding events could not be systematically distinguished.

Iron restriction and anemia may occur through hepcidin-independent pathways. These pathways include a direct cytokine-mediated ferroportin downregulation or the sufficiency of baseline hepcidin to maintain iron sequestration once established [15]. Clinically, urinary hepcidin may thus underestimate subtle regulatory changes in stable elderly individuals, whereas serum hepcidin more accurately reflects the dynamic response in acutely inflamed, hospitalized patients [15, 36]. This was confirmed in the present study, and a moderate association with CRP was additionally demonstrated, indicating that inflammation-driven regulation of hepcidin remains intact even in frail, hospitalized older adults [37, 38].

A weak positive correlation was observed between hepcidin and hemoglobin in the total cohort, but it was no longer statistically significant after stratification by comorbidity and frailty, suggesting that the overall link may reflect heterogeneity in underlying anemia etiologies.

Without concurrent measures of iron status, it is not possible in the present study to distinguish iron deficiency, anemia of inflammation, or mixed etiologies. Iron deficiency and blood loss are generally associated with low hepcidin levels, whereas anemia of inflammation commonly is associated with high hepcidin levels — both conditions leading to reduced hemoglobin, but through opposing regulatory mechanisms [39, 40] .

Figure 3 visualizes that extreme cases with very low hemoglobin levels are associated with low hepcidin levels, which is consistent with previous studies [40]. Although suppression of hepcidin has been observed in the context of severe anemia, no overall linear association between anemia status and hepcidin was evident in the cohort. This pattern is compatible with the coexistence of divergent underlying causes of anemia, where iron deficiency lowers, and inflammation elevates hepcidin levels.

In the present study, no clear association between higher hepcidin levels and anemia was observed. This finding is consistent with previous reports in hospitalized internal medicine patients, where anemia is often multifactorial and not always directly explained by iron deficiency alone [13]. In mild to moderate anemia, inflammatory activation particularly through IL-6 may elevate or maintain hepcidin levels, thereby masking the expected suppression seen in classical iron deficiency [13, 37]. This may be compatible with a state of functional iron deficiency, in which iron availability for erythropoiesis is reduced despite preserved iron stores. In more severe iron depletion, the physiological drive to increase erythropoiesis may overcome inflammatory signaling, leading to secondary hepcidin suppression to restore iron availability [13]. These bidirectional regulatory effects may explain why a stronger linear relationship between hepcidin and hemoglobin was not observed. This interpretation is supported by multivariable analyses in which hepcidin, inflammatory markers, renal function, frailty, and comorbidity were associated with hemoglobin.

Such regulatory complexity may be particularly relevant in older and frail populations, where chronic low-grade inflammation inflammaging and multimorbidity are common. Studies have shown that IL-6 and other proinflammatory cytokines are inversely related to hemoglobin and muscle function, supporting inflammation-driven hepcidin upregulation as a shared mechanism underlying both anemia and frailty. In the meta-analysis by Palmer et al., older adults with anemia had more than twice the odds of being frail compared with non-anemic peers, even after adjusting for comorbid conditions [12]. The context-dependent nature of hepcidin regulation underscores that its interpretation in older or hospitalized patients requires concurrent assessment of iron indices, inflammatory markers, and erythropoietic activity rather than reliance on hepcidin alone.

Hepcidin levels were slightly lower in females (Fig. 4), but this was within expected physiological variation. Levels also increased with decreasing kidney function, consistent with impaired renal clearance as previously reported [8, 14, 25, 35]. Although some variation in hepcidin levels across age, sex, frailty, renal function, and comorbidity was observed, only inflammation-related variables showed strong correlations with hepcidin. Frailty was significantly associated with lower hepcidin levels. Similarly, patients with moderate comorbidity had modestly lower hepcidin levels compared to those with low comorbidity [36].

Differences in admission thresholds between frail and non-frail individuals may influence observed biomarker patterns, although this interpretation remains speculative. This distinction is important because both hepcidin and anemia are associated with acute illness, frailty, and chronic comorbidities [41, 42], and the type of population might be one of the reasons for the observed association between frailty and low hepcidin. Stratified analyses by frailty and comorbidity showed no significant associations between hepcidin and hemoglobin. To investigate these relationships further, studies in population-based cohorts or longitudinal studies can be useful. These findings are consistent with previous literature indicating that inflammatory activity is more strongly associated with hepcidin levels than age, frailty, or comorbidity [4, 36].

### Strengths and limitations

The strengths of this study include the design of a large, real-life cohort, the inclusion of old and frail patients, who are rarely included in clinical trials, up-to-date laboratory evaluation, and blood tests. Some limitations should be pointed out; the cross-sectional design is only a snapshot in time of hepcidin regulation and limits any assessment of temporal dynamics or causal relationships. It was not possible to ascertain the range over which the level of hepcidin is changing with time according to variations in clinical conditions like inflammation, anemia, or frailty. Selection bias might play a role because only acutely hospitalized older patients formed the cohort. Nonetheless, the included patients are expected to be representative of the acutely admitted old patients in Copenhagen, but not representative of the total population of older people in Copenhagen. The multivariable analyses should be interpreted with caution. Given the cross-sectional design and the substantial interdependence between key variables such as inflammation, renal function, frailty, and comorbidity, the models are subject to collinearity and potential residual confounding. The analysis is considered exploratory and do not imply causal relationships. Lastly, no analyses on iron status, such as ferritin, transferrin saturation, MCV or soluble transferrin receptor were measured and compared to the hepcidin levels, which limits interpretation of anemia etiology and erythropoietic regulation.

## Conclusion and perspectives

Hepcidin was found to correlate with the inflammatory markers CRP and IL-6, indicating that inflammation drives increased hepcidin secretion, a mechanism that appears preserved in older and frail patients. In multivariable analyses, the relationship between hepcidin and hemoglobin appeared more complex, reflecting the interplay between inflammation, renal function, and comorbidity. Longitudinal and population-based studies are needed to clarify the impact of hepcidin on hemoglobin levels and to evaluate its potential as a biomarker for anemia subtyping and chronic disease management.

## Data Availability

The datasets used and analysed during the current study are available from the corresponding author on reasonable request.

## Appendix

### Appendix 1

**Table:**

Diagnose category	Diagnose groups
Cardiovascular diseases	Aortic aneurysm; Cardiac arrhythmias; Heart failure; Ischaemic heart disease; Orthostatic hypotension; Peripheral arterial disease (claudication / large-vessel disease); Pulmonary embolism
Digestive diseases	Constipation; Gastroenteritis and diverticulitis; Hernia; Ileus and intestinal perforation; Liver cirrhosis, ascites and hepatic encephalopathy; Lower gastrointestinal bleeding; Non-infectious diarrhoea; Upper gastrointestinal conditions
Endocrine and nutritional diseases	Acute kidney injury and dehydration; Diabetes mellitus; Mild electrolyte disorders; Rhabdomyolysis; Severe electrolyte disorders; Thyroid and pituitary disorders
Hematological diseases	Anaemia; Cytopenia / cytosis
Infectious diseases	Abdominal or thoracic abscess; Bacteraemia; Bronchitis and upper respiratory tract infections; Deep bone and soft tissue infection; Endocarditis; Erysipelas; Gastroenteritis and diverticulitis; Infection of the abdomen, biliary tract and pancreas; Infection of unknown focus; Other uncomplicated infections; Pneumonia; Sepsis; Urinary tract infection
Mental and behavioral disorders	Acute psychosis or delirium; Alcohol intoxication, substance abuse, and drug poisoning; Anxiety, depression, stress-related disorders, and self-harm
Musculoskeletal diseases	Arthritis; Back pain; Concussion; Fracture requiring surgical intervention; Fractures and soft tissue injuries not requiring surgery; Traumatic intracranial haemorrhage
Neurological diseases	Other neurological disorders; Stroke and transient cerebral ischaemia; Undifferentiated neurological symptoms
Other	Adverse drug reaction; Dermatitis; Non-specific functional decline; Suspected malignancy; Vasculitis and pericarditis
R diagnoses (symptoms & abnormal findings)	Abdominal pain; Confusion; Dizziness; Dysphagia; Dyspnoea and cough; Fever; Haemoptysis; Nausea and vomiting; Oedema tendency; Other uncomplicated R diagnoses; Social admission with need for home care; Syncope; Tendency to falls; Unintentional weight loss; Urinary retention
Respiratory diseases	Asthma exacerbation; Bronchitis and upper respiratory tract infections; COPD exacerbation; Pleural effusion; Pneumothorax and respiratory failure
Z diagnoses (factors influencing health status)	Observation for suspected disease; Social admission with need for home care

Diagnosis categories and diagnosis groups. The table provides an overview of the diagnostic categories used in the study and the specific diagnosis groups included within each category

### Appendix 2

**Table:**

Comorbidity Group	Frailty CFS ≥ 5	Non Frail CFS < 5
High Comorbidity (CCI: 9–14), *N* = 85 (8.2%)	46	39
Moderate Comorbidity (CCI: 5–8), *N* = 512 (49.6%)	301	211
Low Comorbidity (CCI: 1–4), *N* = 435 (42.2%)	152	283

Distribution of patients across comorbidity and frailty groups. The majority of patients were classified with moderate comorbidity. The high comorbidity and low frailty group had the smallest sample size (*n* = 39), which may limit statistical power in subgroup analyses

### Appendix 3

**Table:**

Variable	Parameter Estimate	Standard Error	Pr > |t|	Standardized β
Intercept	7.03893	0.17351	< 0.0001	0
Hepcidin log^10^	0.66666	0.06399	< 0.0001	0.35339
Log CRP	-0.10950	0.02876	0.0001	-0.14107
Log il_6	-0.20040	0.03822	< 0.0001	-0.19383
eGFR	0.00937	0.00174	< 0.0001	0.15667
CFS	-0.27287	0.07944	0.0006	-0.10047
CCI	-0.27741	0.06399	< 0.0001	-0.12773

Parameter estimates for the multivariable model with standard errors, *P* value, and Standardized β

## Abbreviations

CRP: C-reactive protein
IL-6: Interleukin-6
eGFR: Estimated glomerular filtration rate
CCI: Charlson Comorbidity Index
CFS: Clinical Frailty Scale
WHO: World Health Organization
SD: Standard deviation
IQR: Interquartile range
LDH: Lactate dehydrogenase
CKD-EPI: Chronic Kidney Disease Epidemiology Collaboration
ICD-10: International Classification of Diseases, Tenth Revision
PROTECT: Prospective Older Patient Evaluation Copenhagen Trial
Hb: Hemoglobin
